# Supplementary material for: Novel high throughput 3D ECM remodeling assay identifies MEK as key driver of fibrotic fibroblast activity
Source: Mater Today Bio. 2025 Apr 24;32:101800. doi: 10.1016/j.mtbio.2025.101800 (PMC12059351; doi:10.1016/j.mtbio.2025.101800)
Supplement: Multimedia component 1 [file mmc1.pdf]

High throughput 3D ECM remodeling assay identifies MEK as key driver of fibrotic fibroblast activity

Chen-Yi Liao[1], Jasmijn H.M. Hundscheid[1], Justin Crawford[2], Peter ten Dijke[3], Beatrice Coornaert[2], Erik HJ Danen[1,4]

[1]Leiden Academic Center for Drug Research, Leiden University, Leiden, the Netherlands; [2]Galapagos NV, Mechelen, Belgium; [3]Oncode Institute and Department of Cell and Chemical Biology, Leiden University Medical Center, Leiden, the Netherlands.

[4]Correspondence to Erik HJ Danen, e.danen@lacdr.leidenuniv.nl

Supplementary Data

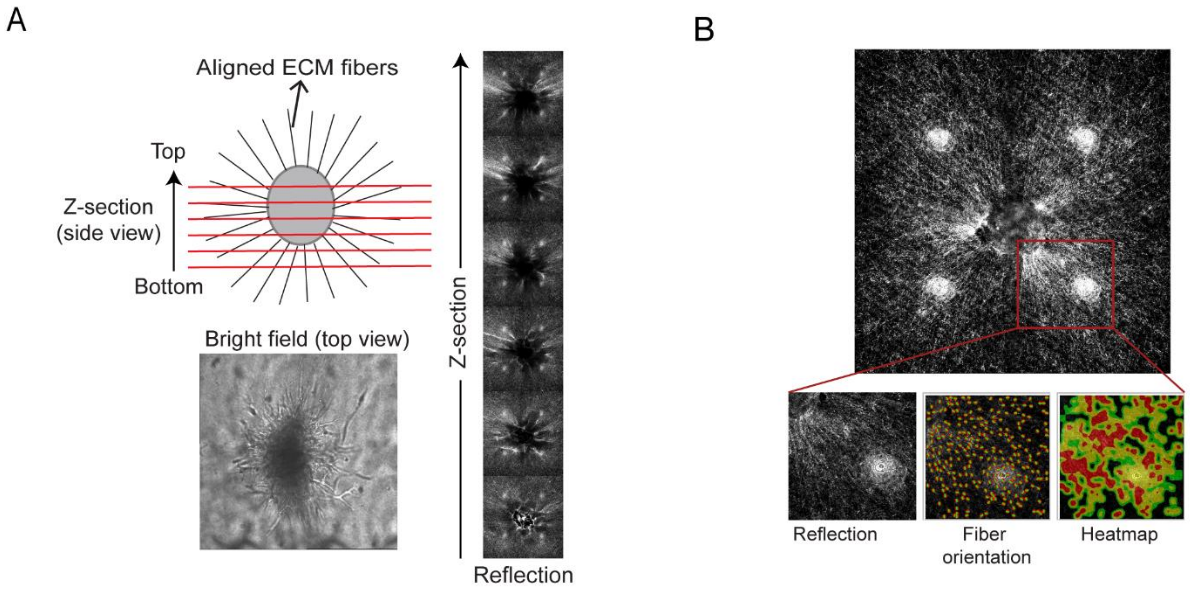

Figure S1. Single cluster model for quantitative assessment of TGFβ- and S1P-induced ECM remodeling. **(A)** Cartoon and brightfield and reflection microscopy images showing aligned ECM fibers perpendicular to the fibroblast cluster measured by reflection microcopy. Z-stack on the right shows reflection images captured at 25 μm vertical spacing throughout the z-axis of a fibroblast cluster. **(B)** Reflection image shows 250 x 250 pixel ROIs in each corner of each Z-section that are selected for analysis using the 'CurveAlign' software [37]. Each ROI image generates two graphical outputs: fiber orientation and heatmap for calculating the alignment index and quantifying the density of aligned collagen fibers, respectively. Note the white spot in each ROI that is an artefact caused by reflection from optical elements in the microscope [36] but which is ignored by CurveAlign as it contains no aligned fibers.

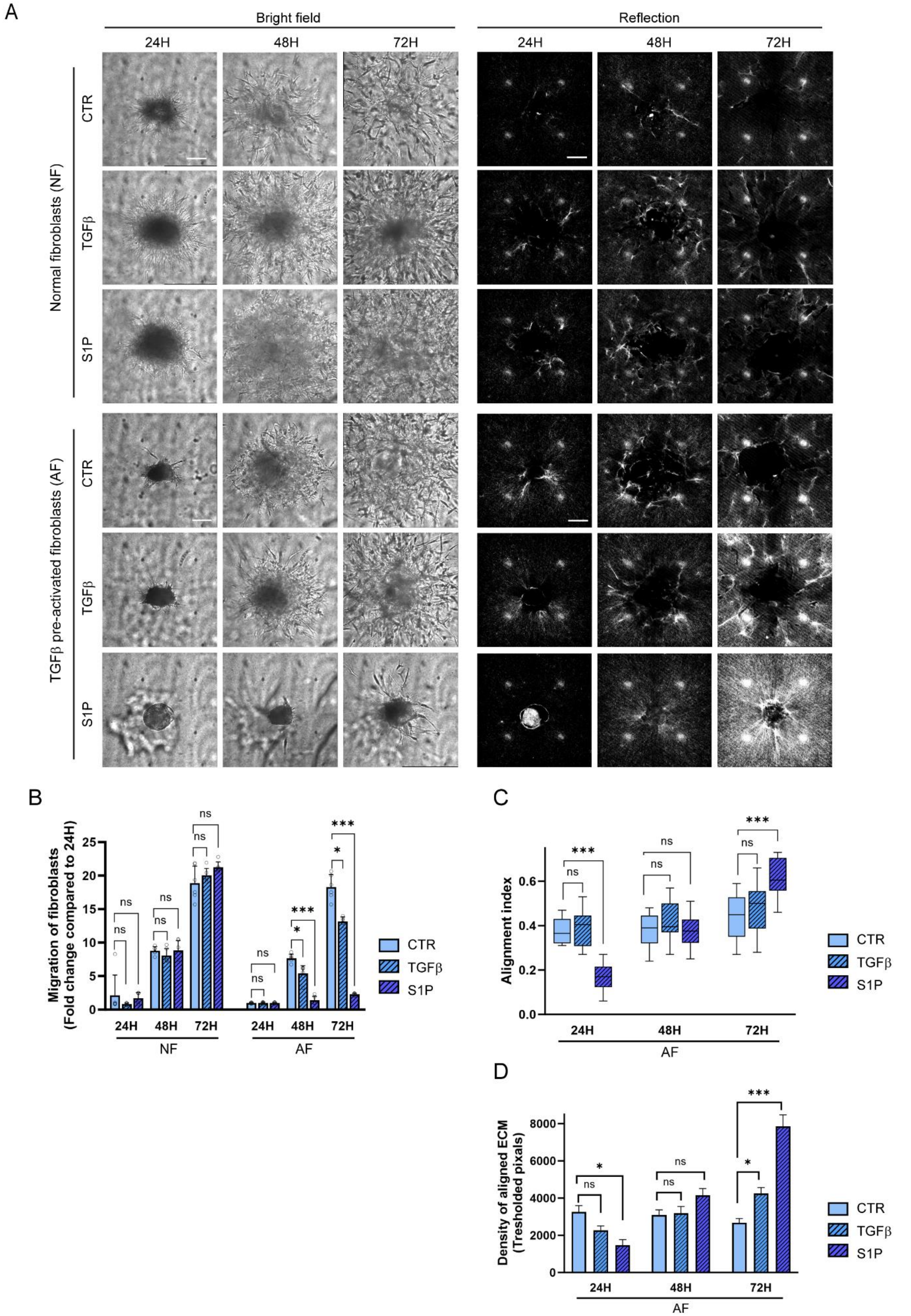

Figure S2. TGFβ and S1P induced ECM remodeling of AF in the single cluster model. (A) Brightfield and reflection (single Z-section) images showing outgrowth and ECM remodeling by clusters of NF or AF

exposed to TGFβ or S1P for the indicated times. Bar = 100 μm. **(B)** Quantification of fibroblast migration based on brightfield images as shown in (A). **(C,D)** CurveAlign based quantification of reflection signals providing fiber alignment index (C) and the density of aligned fibers (D). An alignment index of 1 indicates a high degree of alignment, while an alignment index of 0 indicates a random distribution of fibers. Graphs show the mean and SEM of two independent experiments, each performed in duplicate. Two-way ANOVA followed by Dunnett's multiple comparisons test was performed; NS, non-significant; *, $p<0.05$; ***, $p < 0.001$.

A

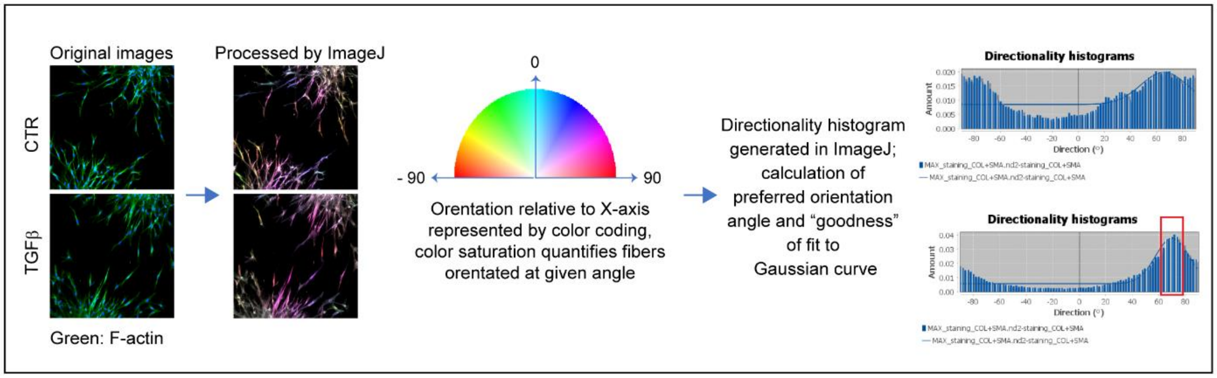

B

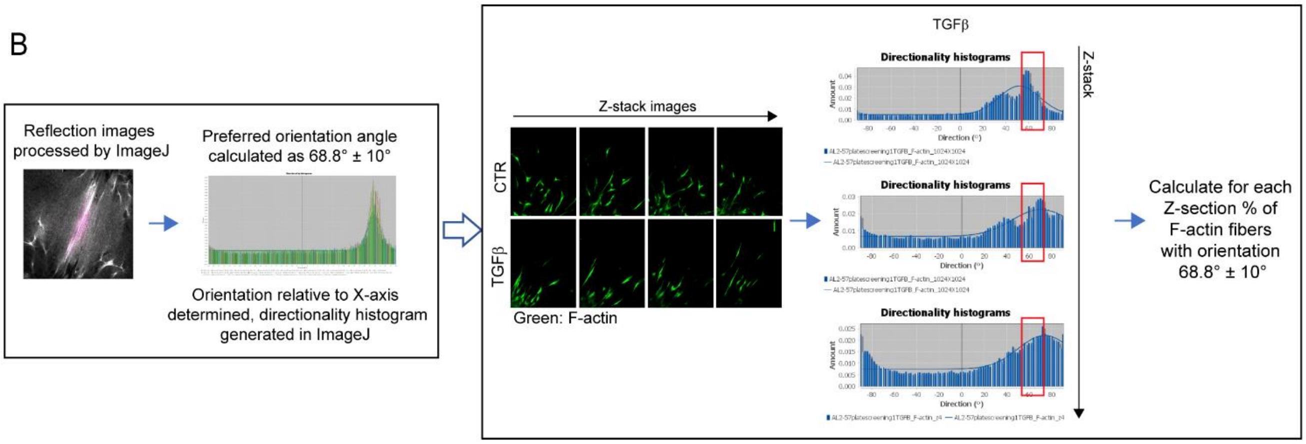

Figure S3. Determination of orientation of cells protruding from fibroblast clusters relative to remodeled ECM. (A) Steps taken for calculation of preferred angle of protruding cells based on F-actin staining. **(B)** Steps taken for calculation of % of cells with an orientation equal to the angle of the ECM bridge.

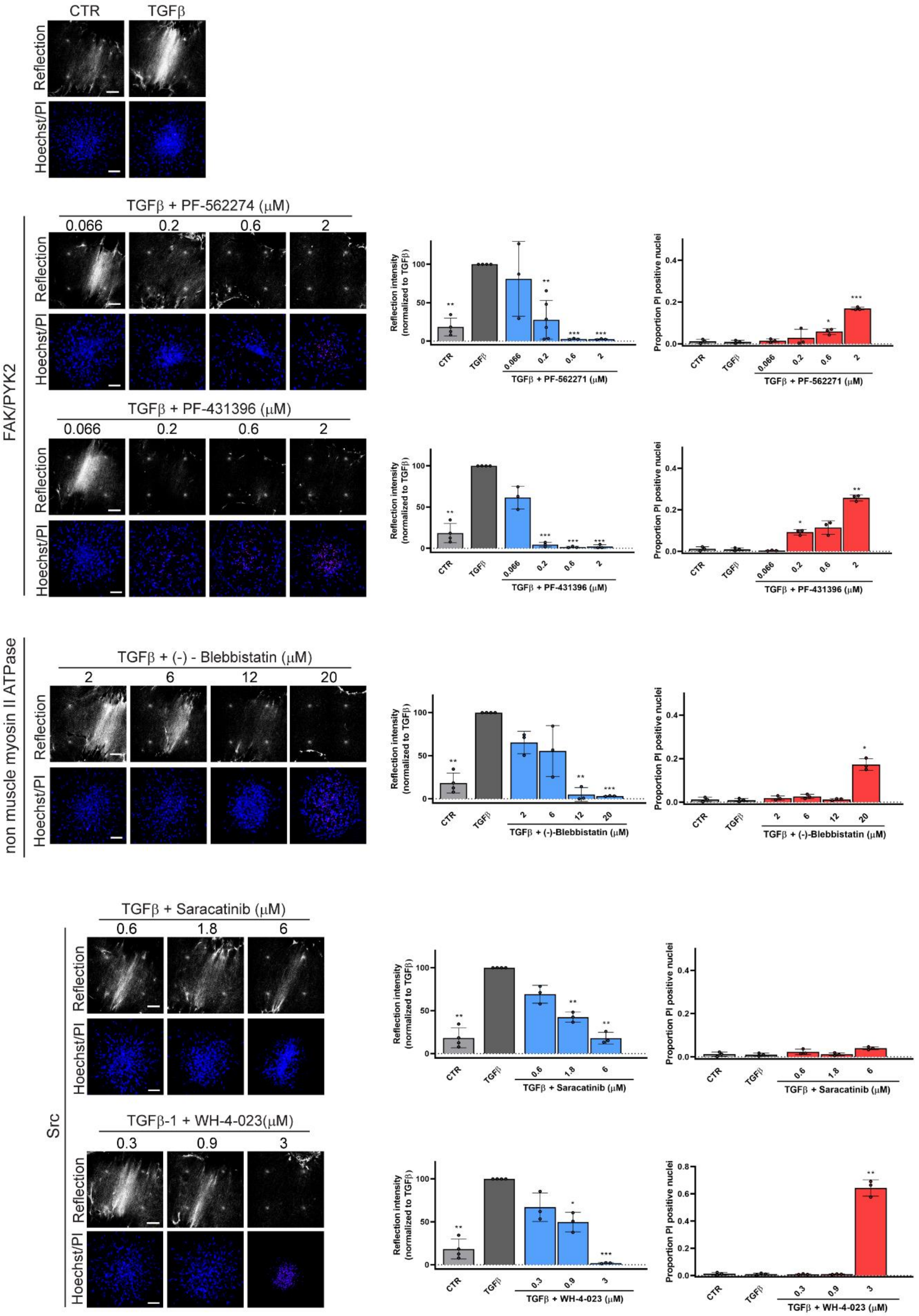

CTR
TGFβ
Reflection
Hoechst/PI
FAK/PYK2
TGFβ + PF-562274 (μM)
0.066
0.2
0.6
2
Reflection intensity (normalized to TGFβ)
Proportion PI positive nuclei
TGFβ + PF-562271 (μM)
TGFβ + PF-431396 (μM)
non muscle myosin II ATPase
TGFβ + (-) - Blebbistatin (μM)
2
6
12
20
TGFβ + (-)-Blebbistatin (μM)
Src
TGFβ + Saracatinib (μM)
0.6
1.8
6
TGFβ-1 + WH-4-023(μM)
0.3
0.9
3
TGFβ + WH-4-023 (μM)

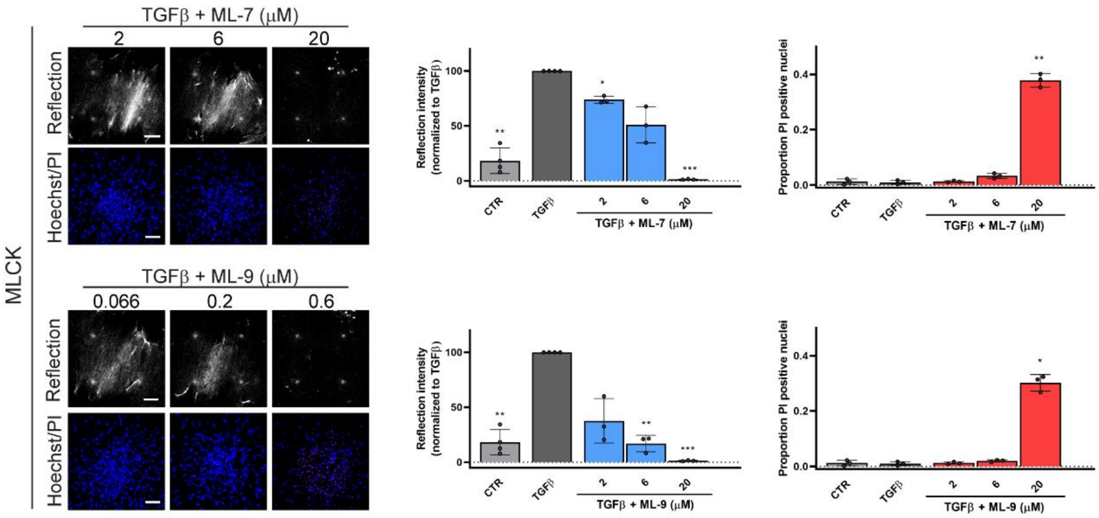

Figure S4. Overview of the efficacy and cytotoxicity of candidate anti-fibrotic compounds. Reflection (single Z-section) images of the area between AF clusters and Hoechst (blue)/PI (red) staining (maximum projection) inside AF clusters exposed for 72 hours to TGFβ containing media in absence or presence of the indicated concentrations of the indicated compounds targeting FAK/PYK2, non-muscle myosin II ATPase, Src, and MLCK. Bar = 100 μm. Graphs show quantification of reflection signals and Hoechst/PI staining. Mean and SEM of two independent experiments, each performed in triplicate is shown. Two-way ANOVA followed by Dunnett's multiple comparisons test was performed; *, $p < 0.05$; **, $p < 0.01$; ***, $p < 0.001$ compared to TGFβ alone condition for reflection and compared to CTR condition for PI staining.

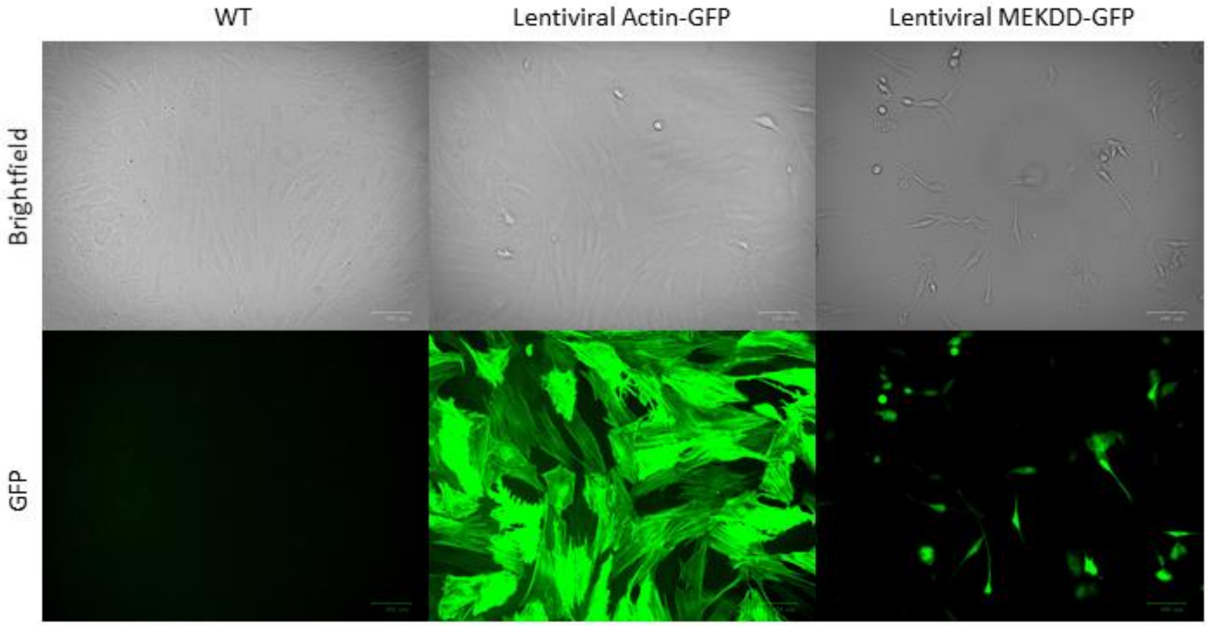

Figure S5. Lentiviral expression of constitutively active MEK leads to cell death in primary human fibroblasts. Brightfield and GFP fluorescence microscopy for primary human fibroblast cultures under control condition of transduced with lentiviral particles expressing the indicated constructs.
